# Discriminating woody species assemblages from National Forest Inventory data based on phylogeny in Georgia

**DOI:** 10.1002/ece3.11569

**Published:** 2024-07-23

**Authors:** Alexander Wellenbeck, Lutz Fehrmann, Hannes Feilhauer, Sebastian Schmidtlein, Bernhard Misof, Nils Hein

**Affiliations:** ^1^ Systematic Zoology University of Bonn Bonn Germany; ^2^ Forest Inventory and Remote Sensing University of Göttingen Göttingen Germany; ^3^ Remote Sensing Centre for Earth System Research (RSC4Earth) Leipzig University Leipzig Germany; ^4^ Institute of Geography and Geoecology Karlsruhe Institute of Technology (KIT) Karlsruhe Germany; ^5^ Leibniz Institute for the Analysis of Biodiversity Change (LIB) Museum Koenig Bonn Germany

**Keywords:** beta diversity, community discrimination, dissimilarity, diversity monitoring, National Forest Inventory, phylogeny, Georgia, unsupervised clustering

## Abstract

Classifications of forest vegetation types and characterization of related species assemblages are important analytical tools for mapping and diversity monitoring of forest communities. The discrimination of forest communities is often based on β‐diversity, which can be quantified via numerous indices to derive compositional dissimilarity between samples. This study aims to evaluate the applicability of unsupervised classification for National Forest Inventory data from Georgia by comparing two cluster hierarchies. We calculated the mean basal area per hectare for each woody species across 1059 plot observations and quantified interspecies distances for all 87 species. Following an unspuervised cluster analysis, we compared the results derived from the species‐neutral dissimilarity (Bray‐Curtis) with those based on the Discriminating Avalanche dissimilarity, which incorporates interspecies phylogenetic variation. Incorporating genetic variation in the dissimilarity quantification resulted in a more nuanced discrimination of woody species assemblages and increased cluster coherence. Favorable statistics include the total number of clusters (23 vs. 20), mean distance within clusters (0.773 vs. 0.343), and within sum of squares (344.13 vs. 112.92). Clusters derived from dissimilarities that account for genetic variation showed a more robust alignment with biogeographical units, such as elevation and known habitats. We demonstrate that the applicability of unsupervised classification of species assemblages to large‐scale forest inventory data strongly depends on the underlying quantification of dissimilarity. Our results indicate that by incorporating phylogenetic variation, a more precise classification aligned with biogeographic units is attained. This supports the concept that the genetic signal of species assemblages reflects biogeographical patterns and facilitates more precise analyses for mapping, monitoring, and management of forest diversity.

## Graphical abstract

For comparison, we applied unsupervised clustering based on a species‐neutral, and dissimilarity index which incorporates interspecies phylogenetic variation to the National Forest Inventory data from Georgia (South Caucasus, *n* = 1059). Our study shows that dissimilarities that consider phylogenetic variation yield more coherent and comprehensible results when classifying species assemblages.

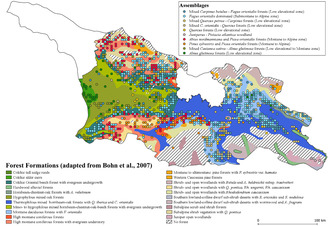

## აბსტრაქტული

ტყის მცენარეულობის ტიპების კლასიფიკაცია და მონათესავე სახეობათა შეკრების დახასიათება მნიშვნელოვანი ანალიტიკური ინსტრუმენტებია ტყის ტიპების აღწერისა და მრავალფეროვნების მონიტორინგისთვის. ტყის ტიპების განსხვავება ხშირად ემყარება β‐მრავალფეროვნებას, რომლის რაოდენობრივი დადგენა შესაძლებელია მრავალი ინდექსის მეშვეობით ნიმუშებს შორის კომპოზიციური განსხვავებულობის გამოსათვლელად. ეს კვლევა მიზნად ისახავს შეაფასოს საქართველოს ეროვნული ტყის ინვენტარიზაციის ზედამხედველობის გარეშე კლასიფიკაციის გამოყენებადობა ორი კლასტერული იერარქიის შედარების გზით. ჩვენ გამოვთვალეთ საშუალო ბაზალური ფართობი ჰექტარზე თითოეული მერქნიანი სახეობისთვის 1059 ნაკვეთზე დაკვირვებით და რაოდენობრივად დავადგინეთ სახეობათაშორისი მანძილი 87‐ვე სახეობისთვის. ჩვენ შევადარეთ სახეობების ნეიტრალური განსხვავებულობიდან მიღებული შედეგები (ბრეი‐კურტისი) ზვავის დისკრიმინაციული განსხვავებულობის საფუძველზე, რომელიც აერთიანებს სახეობათაშორის ფილოგენეტიკურ ვარიაციებს. გენეტიკური ცვალებადობის ჩართვამ განსხვავებულობის რაოდენობრივ განსაზღვრებაში გამოიწვია მერქნიანი სახეობების შეკრების უფრო ნიუანსური განსხვავება და გაზრდილი კლასტერული თანმიმდევრულობა. ხელსაყრელი სტატისტიკა მოიცავს მტევანთა საერთო რაოდენობას (23 v. 20), საშუალო მანძილს მტევნის შიგნით (0.773 vs. 0.343) და კვადრატების ჯამის ფარგლებში (344.13 vs. 112.92). განსხვავებებიდან მიღებული კლასტერებმა, რომლებიც ითვალისწინებენ გენეტიკურ ვარიაციებს, აჩვენეს უფრო მძლავრი გასწორება ბიოგეოგრაფიულ ერთეულებთან, როგორიცაა სიმაღლე და ცნობილი ჰაბიტატები. ჩვენ ვაჩვენებთ, რომ სახეობების შეკრების უკონტროლო კლასიფიკაციის გამოყენებადობა ფართომასშტაბიანი ტყის ინვენტარიზაციის მონაცემებზე მტკიცედ არის დამოკიდებული განსხვავებულობის ფუძემდებლური რაოდენობრივი განსაზღვრაზე. ჩვენი შედეგები მიუთითებს, რომ ფილოგენეტიკური ვარიაციით, უფრო ზუსტი კლასიფიკაციაა შესაძლებელი, რომელიც შეესაბამება ბიოგეოგრაფიულ ერთეულებს. ეს ამტიცებს კონცეფციას, რომ სახეობათა შეკრების გენეტიკური სიგნალი ასახავს ბიოგეოგრაფიულ ნიმუშებს და ხელს უწყობს ტყის მრავალფეროვნების აღწერას მონიტორინგისა და მართვის უფრო ზუსტ ანალიზს.

## 1 INTRODUCTION

Forest ecosystems host the largest share of terrestrial biodiversity and cover approximately one‐third of the global land surface (FAO, 2020; Gillerot et al., 2021; Heym et al., 2021; Torresani et al., 2019). In light of increasing pressure on forests due to climate change and the related global loss of biodiversity, also referred to as the “the sixth mass extinction,” with up to 92% of terrestrial endemics being anticipated to be negatively impacted (Manes et al., 2021), reliable approaches to assess and monitor forest diversity are required (Barnosky et al., 2011; Cowie et al., 2022; Faith, 2013; Palombo, 2021). Monitoring should include the quantification of metrics that allow the classification of ecological entities based on their specific level, or degree of biodiversity, and ultimately according to their respective conservation value, which is required by conservationists (Brooks et al., 2015; Zampiglia et al., 2019). Appropriate delineation of forest communities and characterization of related species assemblages across taxonomic groups are important analytical tools for sensible monitoring of species diversity and biodiversity management. On broader scales, such metrics can be provided by means of vegetation classification which aims to group spatial or temporal diversity of species within a finite set of abstract categories (de Cáceres et al., 2015). Vegetation classification has proven to provide adequate means for descriptive reporting, communication, and mapping of forest communities, and related concepts have responded to changing information needs over time. Consequently, forest‐type classifications exist for a wide range of targets, that is, habitat quality (qualitative assessments for biodiversity management), development over time (i.e., stand classification according to age classes for forest management) or along biogeographic gradients, and remote sensing‐based mapping of ecological communities (de Cáceres et al., 2013; de Cáceres, Martín‐Alcón, et al., 2019; Fassnacht et al., 2016; Hao et al., 2021).

One approach for classifying forest communities focuses on the variation in species compositional characteristics across assemblages of different sites within a geographic area, which is commonly known as β‐diversity (Legendre & de Cáceres, 2013; Magurran & McGill, 2011). β‐diversity can be assessed by the change in species compositional characteristics between sites (i.e., species turnover, Jost, 2010) and a plethora of metrics exist to quantify the degree of dissimilarity between assemblages on various spatial and temporal scales (de Cáceres et al., 2013; de Cáceres, Coll, et al., 2019; Legendre & Legendre, 2012; Magurran & McGill, 2011; Ricotta, 2005). The most common dissimilarity indices are exclusively based on compositional characteristics, that is, species richness and elements of evenness (Magurran, 2005), while interspecies variability (i.e., phylogenetic, taxonomic, functional, or traits) is not considered (Chao et al., 2018; Chiu et al., 2014; de Cáceres et al., 2013; Hao, Ganeshaiah, et al., 2019; Pavoine et al., 2013). In line with the increasing recognition that genetic diversity comprises an integral part of biodiversity, for example, as stated in the definition of biodiversity by the Intergovernmental Platform on Biodiversity and Ecosystem Services (IPBES, Díaz et al., 2015), literature on how to incorporate phylogenetics as aspect of diversity is growing rapidly (Chao et al., 2023). Accordingly, several authors have approached forest community classification by accounting for both compositional data and interspecies phylogenetic variability (i.e., Capelo, 2020; Hao et al., 2021; Ricotta et al., 2020; Webb et al., 2002). As phylogenetically closely related species often share beneficial traits for specific environments, discriminating assemblages based on phylogenetic distances can serve as a proxy for classifying forest communities according to functional roles, environmental diversity, and conservation value (Faith, 2013; Gilbert & Parker, 2022; Hawkins et al., 2014; Padullés Cubino et al., 2021; Pavoine, 2016; Pavoine & Ricotta, 2014; Tucker et al., 2017). Hao, Ganeshaiah, et al. (2019) demonstrated that different patterns emerged if interspecies taxonomic distances were considered for the classification of global forest communities using the Discriminating Avalanche index (Ganeshaiah & Shaankar, 2000).

On a smaller scale, National Forest Inventories (NFIs) provide systematic and periodical observations of tree species abundances based on permanent sample units on a country‐wide level (Corona et al., 2011). The continuous adaptation of variables assessed during NFIs highlights an increasing emphasis on aspects of biodiversity, enabling ecologists to investigate potentials and limitations of the thus provided data (Alberdi et al., 2019; Corona et al., 2011; Didion, 2020; McRoberts et al., 2009). Incorporating phylogenetic diversity of species assemblages extends the perspective on diversity in this context and bears the potential to deepen our understanding of the complex interactions among woody species over large geographical scales.

In the present study, we compare the performance of two dissimilarity indices for the discrimination of forest woody species assemblages when applied to large‐scale forest inventory data such as the dataset of the first NFI of Georgia. To this end, we applied unsupervised clustering to the obtained dissimilarity matrices based on a conventional and a dissimilarity index that incorporates interspecies phylogenetic distances, respectively. Apart from statistics for internal evaluation of the resulting classifications, our comparison considered the distribution of discriminated assemblages along biogeographic gradients. Based on the assumption that genetic variability of co‐inhabiting species provides a signal that sufficiently reflects site‐specific environmental determinants, we investigated whether including this variable in the quantification of dissimilarity results in an improved reflection of biogeographic gradients. To test the general applicability, we incorporated the phylogenetic variability into the classification of a large, real‐world dataset and evaluated the results considering cluster cohesiveness and overall interpretability.

## 2 DATA AND METHODS

We compare two dissimilarity indices for the classification of woody species assemblages when applied to NFI data of Georgia. Adhering to the methodological approach underlying the data, we focus on woody species, that is, all recorded species that meet the specified target diameter at breast height (DBH, at 1.3 m, MEPA, 2018). Consequently, we are referring to woody species even if only species is written hereafter.

### 2.1 Study area

Georgia is located between the Southern Slopes of the Greater and the Northern part of the Lesser Caucasus, between 41°07′ – 43°35′ N and 40°04′ – 46°44′ E (Fischer et al., 2018). The forests of Georgia host large shares of endemic species and form part of the Caucasus biodiversity hotspot (Joppa et al., 2011; Myers, 2003). Existing forest formations range from Alpine coniferous forests dominated by *Abies nordmanniana* (Steven) Spach. and *Picea orientalis* (L.) Peterm. to open juniper woodland (dominated by *Juniperus polycarpos excelsa* subsp. *polycarpos* (K. Koch) Takht. and *J. foetidissima* Willd.), encompassing further thermophilus to xerophytic mixed oak forest (*Quercus petrea* subsp. *iberica* (Steven ex M. Bieb.) Krassiln., *Carpinus betulus* L., and *C. orientalis* Mill.), Colchic alder carrs which are dominated by *Alnus glutinosa* subsp. *barbata* (C. A. Mey.) Yalt. and oriental beech (*Fagus orientalis* Lipsky) and hornbeam‐oriental beech forests (Bohn et al., 2007; Dolukhanov, 2010; Fischer et al., 2018; Nakhutsrishvili et al., 2021; Novák et al., 2023).

### 2.2 Forest community data

**FIGURE 1:**
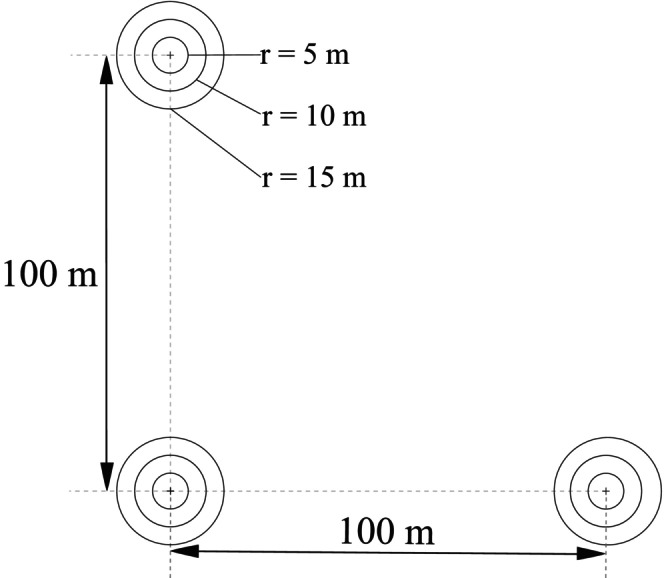
Cluster plot configuration of the first National Forest Inventory of Georgia. Each cluster consists of three subplots for tree assessment within three concentric circles according to the measured diameter at breast height (DBH, at 1.3 m, MEPA, 2023).

**FIGURE 2:**
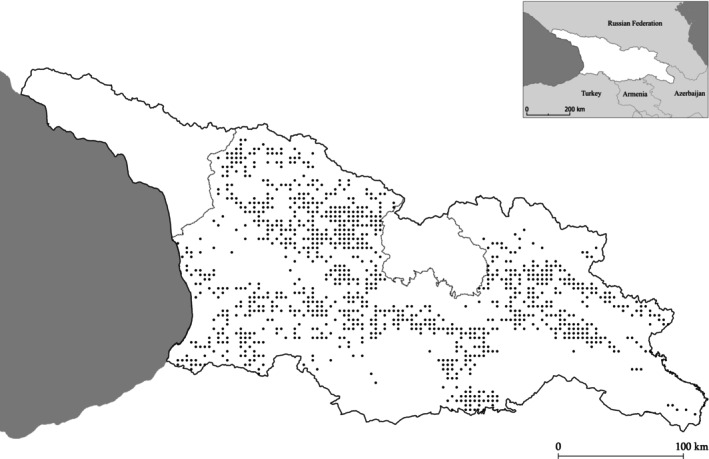
Locations of samples used from the National Forest Inventory of Georgia, *n* = 1059. Samples (black dots) consist of accessible cluster plots of equal sample size (three subplots) located inside forests that are not intersected by forest boundaries and contain only records of taxa identified at species level. Dashed lines mark the boundaries of inaccessible areas from where no field data were obtained.

Between 2018 and 2021, Georgia implemented its' first NFI based on a systematic sampling grid of 3.6 × 3.6 km with a randomly selected origin. Sampling units consist of cluster plots (0.21 ha) containing three subplots of 0.0.7 ha each. These subplots are arranged in an L‐shaped configuration with a distance of 100 m along both axes (Figure 1). As 18% of the country's territory is currently not accessible for government officials due to an ongoing political conflict (MEPA, 2023), clusters were sampled on approximately 74% of the national forest area (Figure 2).

#### 2.2.1 Assessment of Woody species

Woody species were recorded per subplot on three concentric nested subplots according to any stems' respective DBH. Stems with DBH ≥ 30 cm were recorded on the largest plot (*r* = 15 m). Stems with DBH ≥ 15 and ≥8 cm were recorded on the inner nested plot radii of 10 m radius and 5 m, respectively (Figure 1). Numerous variables were recorded per woody species, along with the polar coordinates of the stem axis, species, and DBH (MEPA, 2018).

From the entire NFI data set (*N* = 2006), all accessible clusters pertaining to the locally applied land use class “Forest” and sub‐classes “Tree covered area” or “Fire affected forests” were selected for analysis (MEPA, 2018). Subplots with recorded intersections with a forest boundary (“slopover sample plots”) were excluded to avoid including samples with extreme outliers regarding species richness due to edge effects (Willmer et al., 2022). Clusters containing species observations that were not unambiguously identified at species level, for example, *Deciduous* spp. and *Genera* spp., were omitted because a precise quantification of the cophenetic distances along the phylogenetic hierarchy is not possible. Clusters containing subplots without woody species observations were excluded. Our sample consists of all cluster plots comprised of observations from three subplots (*n* = 1059, henceforward referred to as “samples”), which represent 53% of all clusters. Figure 2 provides an overview of the spatial distribution of samples used for the analysis.

After reprojection of sample locations to UTM38N, WGS84 (EPSG: 32638), sample elevations [m above sea level] were derived from the digital elevation model (DEM) provided by Shuttle Radar Topography Mission (SRTM, Farr et al., 2007). Elevation values were calculated as the median of all raster cell values (30 × 30 m) contained in or crossed by the circular subplot area (*r* = 15 m + recorded GPS error [m]) of the southwestern subplot.

#### 2.2.2 Species diversity data

Diversity measures are usually based on data representing the compositional variation between species (i.e., occurrence and abundance) in an assemblage (Ricotta et al., 2021). Forest species communities may display similar compositional characteristics, in terms of counts of observed species and respective individuals. However, species can be represented by large numbers of small‐diameter trees, or stems belonging to the same individual, or fewer individuals but with significantly large relative shares of total basal area. Hence, abundance estimates based on counts of individuals do not take significant differences in the size structure of occurring species into consideration and may result in distinct evenness profiles. We used mean basal area (BA, m^2^/ha) per species and cluster plot as species abundances to account for the variation in size of the constituents. By weighting compositional data using BA, we incorporate valuable structural information that considers site occupation per species for the quantification of β‐diversity (de Cáceres, Coll, et al., 2019; McRoberts et al., 2009; Staudhammer & LeMay, 2001; Yao et al., 2019). Consequently, BA of all living stems was aggregated per cluster plot and species and divided by 3 to obtain mean BA estimates per sample (de Cáceres et al., 2015; MEPA, 2018).

### 2.3 Analysis

#### 2.3.1 Nomenclature

Spelling and nomenclature of all recorded species were standardized with the Taxonomic Backbone databases of World Flora Online (WFO DB, Kindt, 2020) and the Global Biodiversity Information Facility (GBIF Secretariat, 2021). In cases where species listed in the NFI data did not yield an unequivocal match in the WFO DB, corresponding records were harmonized with Lachashvili et al. (2022) and the nomenclature of the World Plants database (https://www.worldplants.de) to derive names for all species ranked as taxonomically accepted.

#### 2.3.2 Phylogenetic interspecies distances

**FIGURE 3:**
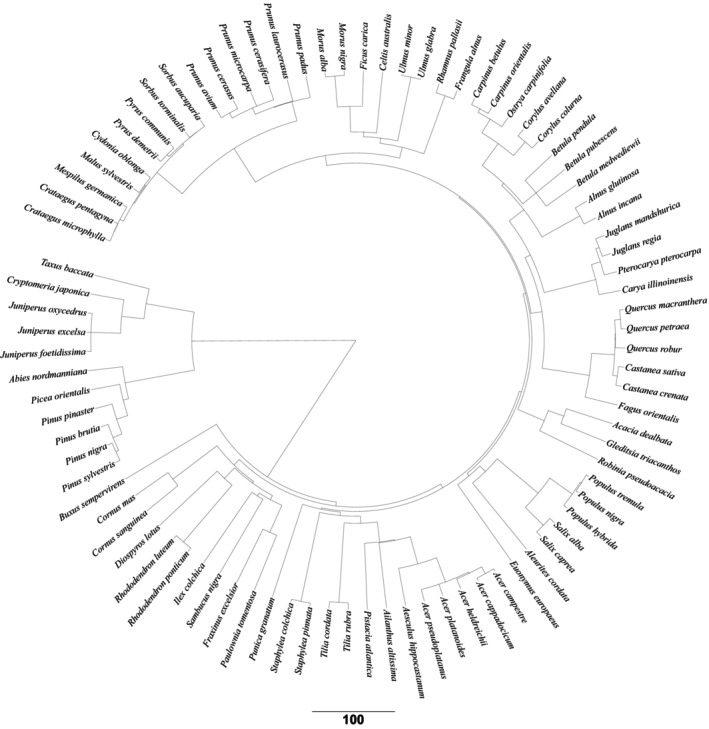
Phylogeny for 87 species listed in the data sample of the National Forest Inventory of Georgia. Interspecies phylogenetic distances were calculated as total branch length connecting each pair of species at the terminal nodes of the hierarchy. For respective branch lengths, see Figure S1.

A phylogenetic tree encompassing all observed species was constructed by matching the harmonized species list with a mega phylogeny containing 72,570 species of vascular plants according to the World Plants database (GBOTB.extended.WP.tre, Jin & Qian, 2022). The backbone mega phylogeny is based on the species‐level phylogeny for vascular plants derived from gene sequencing from 7 gene regions and 39 fossil calibrations created by Zanne et al. (2014), which was updated and expanded by Jin and Qian (2022). Following the authors' recommendation to consolidate taxa below species level (e.g., sub‐species) with the respective parental species, five infraspecific taxa were combined with their parental species, resulting in the lowest taxonomic unit being species level (Figure 3). From the thus created ultrametric phylogenetic tree (Jin & Qian, 2019; Qian & Jin, 2016; Smith & Brown, 2018), cophenetic distances, that is, the total branch length connecting each pair of species at the terminal nodes of the respective phylogeny, were calculated (Bevilacqua et al., 2021; Hao, Corral‐Rivas, et al., 2019; Kling et al., 2018).

Interspecies phylogenetic distances were normalized within a square matrix that contained pairwise distance values [0 < dist_ph_ ≤ 1] between each pair of species.

#### 2.3.3 Dissimilarity indices

The Discriminating Avalanche index (dA equation [2] in Table 1) developed by Ganeshaiah and Shaankar (2000) and described by Hao, Corral‐Rivas, et al. (2019) considers interspecies dissimilarity by multiplying absolute differences in frequencies (numerator in BC) of species *i* and *j* in two samples with a specific distance between species *i* and *j*. We use the phylogenetic distances to weigh the difference in mean basal area between *i* and *j*. Table 1 shows the formulas of both indices used in this study.

**TABLE 1:** Dissimilarity indices used in this study.

Bray–Curtis dissimilarity index (1957)	BC=$\frac{\sum_{i=1}^{n}\left|p_{i}^{a}-p_{i}^{b}\right|}{\sum_{i=1}^{n}\left(p_{i}^{a}+p_{i}^{b}\right)}$	[1]
Discriminating Avalanche (Hao, Corral‐Rivas, et al., 2019)	$\mathrm{dA}=\frac{1}{2}\sum_{i=1}^{n}\sum_{j=1}^{n}{∆}_{i}^{a,b}d_{\mathrm{ij}}{∆}_{j}^{a,b}$	[2]

*Note*: With *d*_*ij*_ = phylogenetic distance between species *i* and *j* (*d*_*ij*_ = *d*_*ji*_ and *d*_*ii*_ = 0); ${∆}_{i}^{a,b}$ = absolute difference between the frequencies of species *i* in plots *a* and *b* $\left(\left|p_{i}^{a}-p_{i}^{b}\right|\right)$; *n* = total number of sample plots; $p_{i}^{a},p_{i}^{b}$ = relative frequencies of species *i* in plots *a* and *b*.

As the maximum dissimilarity value obtained by dA = $\left(1-\frac{1}{n}\right)$, where *n* equals the number of species, the resulting dissimilarities were normalized via $x_{\mathrm{norm}}=\frac{x-x_{\min}}{x_{\max}-x_{\min}}$, with *x*_min_ and *x*_max_ representing the minimum and maximum value of dA, respectively (Hao, Corral‐Rivas, et al., 2019; Legendre & Legendre, 2012). Consequently, pairwise dissimilarity values of the two resulting dissimilarity matrices (1059 × 1059) range between 0 and 1. Pairwise values of 1 imply that two samples are completely different as they do not share any species, whereas values of 0 indicate two samples are equal in terms of compositional characteristics (Chao et al., 2005; Legendre & Legendre, 2012; Leyer & Wesche, 2008). Prior to clustering, a Mantel test (Mantel, 1967) was performed to check for existing correlations between the two dissimilarity matrices. As the pairwise dissimilarities are not normally distributed, and non‐linear relationships between the pairwise dissimilarity values exist, we used the Spearman correlation coefficient with 9999 permutations (Legendre & Legendre, 2012).

#### 2.3.4 Isometric partitioning

The isopam algorithm (Schmidtlein et al., 2010) available in package “isopam” (v. 2.0, Schmidtlein et al., 2024) combines isometric feature mapping and partitioning around medoids (data points that are most centrally located within each cluster with the sum of dissimilarities between medoids and all other data points being minimized) in order to build clusters with a maximum number and fidelity of indicative species. The isomap ordination, which is based on geodesic distances strongly determined by neighborhood definitions, is repeated with different parameter settings. The result is clustered, and the clusters are evaluated according to the criteria mentioned above. These criteria are similar to those used when structuring phytosociological tables (Abe, 2021). In this, and the use of an ordination, isopam is similar to twinspan (Hill, 1979), but does not involve internal readjustments, uses geodesic distances (taking account of “neighbors of neighbors” in feature space), and works on multidimensional ordination spaces. It has been previously used for large‐scale classifications of forests (Cabido et al., 2018; Černý et al., 2015; Zeballos et al., 2020) and other systems (Feilhauer et al., 2021; Hein et al., 2014; Peterka et al., 2017; Yu et al., 2022). isopam can be run both unsupervised and supervised (with reference plots). For the current study, the original source code was extended to support dA (Capelo, 2020) and executed on a computer with two Intel Xeon CPUs (E5‐2630 v3) and 256 GB RAM using R Statistical Software (v 4.3.2; R Core Team, 2023). To ease comparability, we set the maximum number of hierarchy levels to four for both classifications. Subsequently, we extracted lists of indicator species frequencies with levels of significance according to Fisher's exact test for each cluster using the isotab function, which is part of the “isopam” package. Fidelity (“equalized phi,” Tichy & Chitrý, 2006) together with Fisher's exact test if the observed frequency is not attained by chance are the criteria for qualifying as an indicator species in isotab (Schmidtlein et al., 2024).

#### 2.3.5 Evaluation of clustering

To evaluate the correspondence between the original sample dissimilarities and dendrogram distances, we calculated cophenetic correlation coefficients for each hierarchical cluster structure (Lapointe & Legendre, 1995; Legendre & Legendre, 2012). The modified Rand index was used to evaluate clustering performance based on the consistency between partitions (Legendre & Legendre, 2012). Cluster homogeneity was evaluated via within sum of squares (WSS, Hao, Ganeshaiah, et al., 2019) and a comparison of the average distance between and within clusters using the function cluster.stats of the R package “fpc.” To assess relevance of the hierarchies, we compared indicator species and the resulting distributions of relative BA among partitioned groups (de Cáceres et al., 2015). Evaluation of correspondence to biogeographic units was based on a comparison of elevational ranges derived from the DEM between groups and the spatial distribution of clusters in relation to forest vegetation‐type classifications presented by Bohn et al. (2007). We applied the nonparametric Kruskal–Wallis test to check for significance between groups due to nonnormality in the distribution of elevation values within groups (Shapiro test). Henceforward, we are referring to the initial partition at the lowest level of the hierarchy as classes, to the intermediary partitions as branches, and the resulting clusters as assemblages.

#### 2.3.6 Data analysis

The analysis was conducted in R Studio version 2023.09.0‐463 (RStudio Team, 2020) using R Base version 4.3.0 (R Core Team, 2023). Harmonization of nomenclature was realized via the R package “Worldflora” version 1.13‐2 (Kindt, 2020), and the package “V.phylomaker2” version 0.1.0 was used to match observed species with the phylogenetic backbone (Jin & Qian, 2022). The Mantel test and BC dissimilarities were calculated using the packages “vegan” version 2.6‐4 (Oksanen et al., 2022). A custom function was embedded in the adjusted clustering algorithm of the corresponding R package “isopam” version 1.2.0 for dA (Schmidtlein et al., 2022). Clustering metrics were obtained using the R packages “mclust,” version 6.0.0 (Scrucca et al., 2016), and “fpc,” version 2.2‐10 (Hennig, 2023).

## 3 RESULTS

Compositional data of *n* = 1059 samples containing 65,818 living tree observations were analyzed (Table 2). In total, 87 species were represented by 52 genera, 29 families, 16 orders, and two classes.

**TABLE 2:** Summary statistics of compositional data of cluster plot observations of the National Forest Inventory of Georgia.

Species richness					Mean basal area per species [m^2^/ha]			
*n*	Min	Max	Mean^a^	CV%	Min	Max	Mean^a^	CV%
1059	1	12	4.96 (±2.14)	43.08	2.54	79.02	30.4 (±12.79)	42.06

Abbreviations: CV%, Coefficient of variation; Var., Variance.

^a^ Means are denoted with standard deviation in parenthesis.

Angiosperms were represented by 76 species (in 87.4% of all samples) across 26 different families. *Fagaceae* (six species) accounted for the highest number of observations followed by *Betulaceae* with 10 species (25.3% and 23.2% of all samples, respectively), *Rosaceae*, and *Sapindaceae* (15 and 6 species, in 11.4% and 10.3% of all samples, respectively). In contrast, gymnosperms (10.3%) were represented by 11 species, belonging to three different families, with the largest family being *Pinaceae* (6 species) followed by *Cupressaceae* and *Taxacea*e (4 and 1 species, respectively). Gymnosperms were observed in 33% of all samples.

### 3.1 Pairwise dissimilarity

Mantel statistics (*r* = .613 with *p* = .0004) indicated a significant positive correlation between the two dissimilarity matrices. Pairwise dissimilarities according to dA ranged from 0.23 to 0.87, which is almost double the range of that of BC (0.63 to 0.99, Table 3).

**TABLE 3:** Summary statistics of mean pairwise dissimilarities between samples based on Bray–Curtis (BC) and Discriminating Avalanche (dA) of the National Forest Inventory data of Georgia.

Index	*n*	Min	Max	Mean^a^	Var.	CV%
BC	1059	0.627	0.998	0.789 (±0.099)	0.01	12.55
dA		0.225	0.87	0.383 (±0.147)	0.022	38.38

Abbreviations: CV%, Coefficient of variation; Var., Variance.

^a^ Means are denoted with standard deviation in parenthesis.

Consequently, the mean dissimilarity between samples (0.79 for BC and 0.38 for dA, respectively) and thus overall variation was higher for BC than for dA, as for the latter, frequencies of dissimilarity values <1 were more evenly distributed, with very few pairwise dissimilarities of 1. Frequency distributions of sample dissimilarities are provided in Figure S2.

### 3.2 Discrimination of assemblages

**FIGURE 4:**
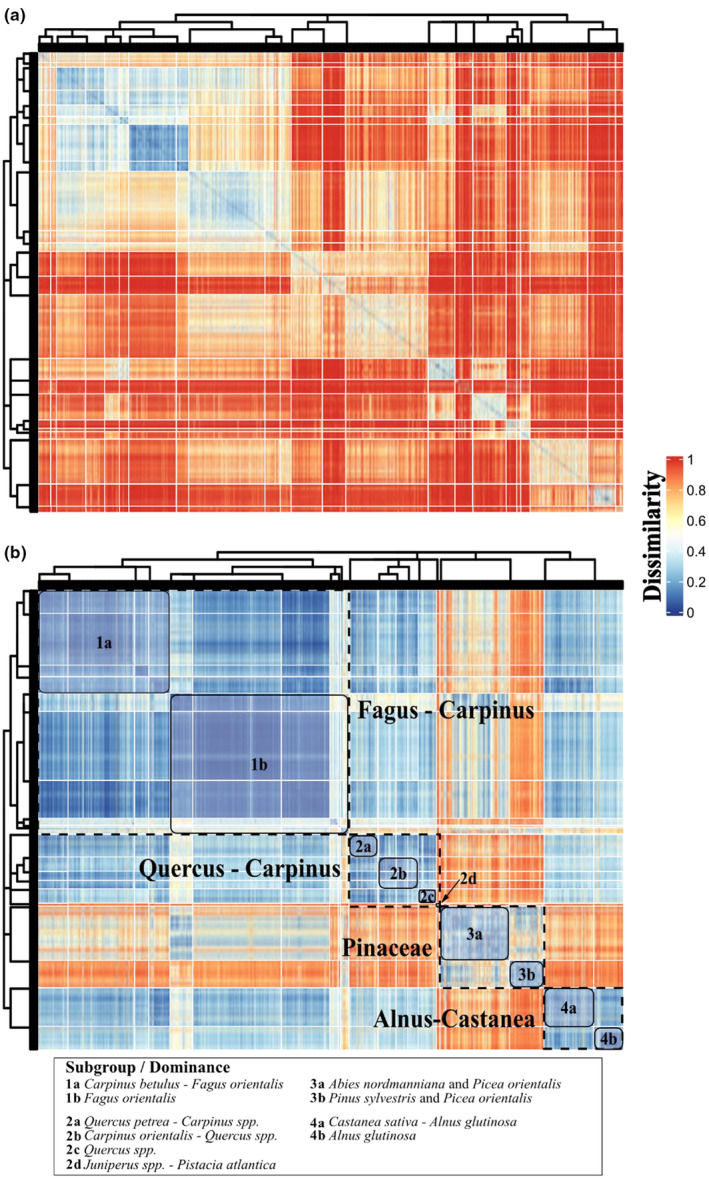
Resulting cluster hierarchy from the isopam partitioning (dendrogram) and pairwise dissimilarities of 1059 samples from the National Forest Inventory data of Georgia. Dissimilarities are based on the Bray–Curtis (a) and discriminating avalanche (b) index. The cell grid is colored according to the dissimilarity values between samples (rows and columns). In total, 23 and 20 assemblages were discriminated for Bray–Curtis and discriminating avalanche, respectively. At level II of the hierarchical clustering based on the discriminating avalanche index, 10 subgroups distributed over four classes were labeled according to dominance of relative basal area.

The hierarchical clustering based on BC (HC_BC_) distinguished 23 assemblages (clusters) across four hierarchical levels (I–IV) and classes. Within HC_BC_, samples were partitioned into 10 and 17 branches at levels II to III, respectively. The hierarchical clustering based on dA (HC_dA_) contained 10 and 15 branches at levels II to III and resulted in 20 distinct assemblages over four levels and classes. Two assemblages were not partitioned further below level II in HC_dA_ (Figure 4). The number of samples per assemblage ranged from 6 to 152 for HC_BC_ (Mean: 82 ± 91.5) and 3 to 163 for HC_dA_ (Mean: 106.1 ± 119.9), respectively. For HC_BC_, the number of observed species per assemblage ranged from 12 to 54. For HC_dA_, one assemblage contained only four species, whereas two assemblages encompassed 53 species.

The cophenetic correlation coefficients were 0.492 and 0.442 for HC_BC_ and HC_dA_, respectively. The obtained adjusted Rand index of 0.317 suggests a modest level of similarity between the clustering results. Average distances within clusters ranged from 0.671 to 0.875 and 0.196 to 0.49 for HC_BC_ and HC_dA_, respectively. Mean distance between clusters was 0.789 for HC_BC_ and 0.384 for HC_dA_. WSS values of HC_BC_ amounted to 344.13 and 112.92 for HC_dA_.

#### 3.2.1 Evaluation of cluster hierarchies

To evaluate the performance of BC and dA for clustering, we compared the resulting classes, groups, and assemblages considering internal metrics of the partitioning process. A total of 77 and 68 indicator species were listed for all partitions based on BC and dA, respectively. For a characterization based on indicators and respective frequencies, only highly significant (*p* ≤ .001) species with total frequencies ≥50% were considered, unless indicated otherwise. Indicator species for both partitions were *A. nordmanniana*, *A. cappadocicum*, *A. glutinosa*, *C. betulus*, *Carpinus orientalis* Mill., *C. sativa*, *F. orientalis*, *P. orientalis*, *Q. petraea* subsp. *polycarpa* (Schur) Raus, and *Tilia rubra* subsp. *caucasica* (Rupr.) V. Engl. In addition, *Fraxinus excelsior* L. is an indicator for HC_dA_. The total number of indicators with frequencies of 100% was 24 and 8 for HC_BC_ and HC_dA_, respectively. Class 1 of HC_BC_ is characterized by a high frequency of *F. orientalis* (99%), whereas for HC_dA_, *F. orientalis* and *C. betulus* are listed with frequencies of 93% and 75%, respectively. In class 2, the highest frequencies are observed for *Q. petrea* (87%) and *C. betulus* (82%). *P. orientalis* (68%), *F. orientalis* (57%), and *A. nordmanniana* (53%) are the most frequent indicator species in class 3, whereas for class 4, these are *A. glutinosa* (87%) and *Castanea sativa* Mill. (61%). In HC_dA_, highly significant indicators in class 1 are *F. orientalis* (93%) and *C. betulus* (75%), whereas in class 2, these are *Q. petrea* (84%), *C. orientalis* (61%), and *Fraxinus excelsior* L. (50%). In classes 3 and 4 of HC_dA_, *P. orientalis* (79%), *A. nordmanniana* (64%), *A. glutinosa* (92%), and *C. sativa* (59%) represent highly significant indicators in classes 3 and 4, respectively. Based on these characteristics, we labeled the four main classes according to predominant relative BA and are referring to these for ease of readability henceforward as follows: class 1 is characterized by a dominance of *Fagus*, class 2 is *Carpinus‐Quercus* dominated, and classes 3 and 4 are *Pinaceae* and *Alnus‐Castanea* dominated, respectively. Synoptic tables of both cluster hierarchies (Figures S3 and S4, respectively) and a detailed description of indicator distributions per partition below level I for HC_dA_ (Appendix S1) are provided as appendices.

#### 3.2.2 Elevation and spatial distribution

**FIGURE 5:**
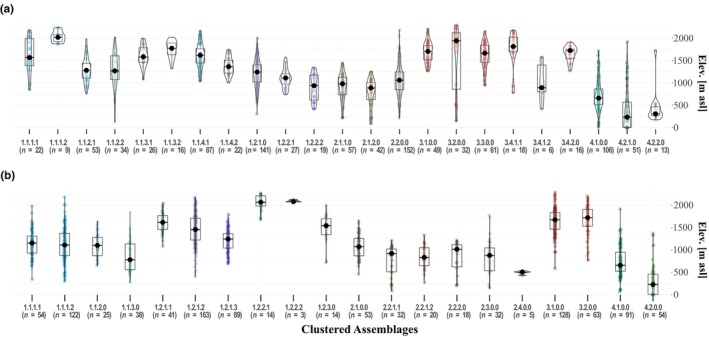
Sample elevations [m asl] from the National Forest Inventory in Georgia per resulting cluster for Bray–Curtis (a) and discriminating avalanche (b). The boxes represent interquartile ranges and respective median values (solid line) of sample elevations within each clustered assemblage (colored dots). Resulting *p* values of the *X* ^2^_Kruskal–Wallis_ test are *p* = 2.899e‐113 and *p* = 1.674e‐115, with confidence intervals between 0.544, 1 and 0.538, and 1 for assemblages in the hierarchical clustering based on BC and dA, respectively (*n* = 1059).

To evaluate the correspondence of assemblages to existing biogeographic units, we compared the distribution of sample elevations within assemblages. Within the four classes in both hierarchies, sample elevations are distributed similarly. Samples within the *Fagus*‐dominated group (class 1) cover a wide elevational range (≤750 m to >2000 m asl), however, in HC_BC_, 81% of all samples are located between >1000 and 2000 m asl, whereas in HC_dA_, most samples (77%) are located within the lower range of >750 and 1750 m asl. Samples with *Carpinus‐Quercus* dominance are predominantly located at elevations <1250 m in both hierarchies. The majority of *Pinaceae*‐dominated samples lie above 1250 m asl, whereas all of *Alnus‐Castanea*‐dominated samples are situated below 1250 m asl, with the majority (70%) positioned below 750 m asl for HC_BC_ and HC_dA_, respectively. Overall classes, except for the *Fagus*‐dominated group, agglomerations of sample elevation values are more pronounced within assemblages of HC_dA_ than of HC_BC_. The applied Kruskal–Wallis test revealed highly significant differences (*p* ≤ .001) between assemblages for both hierarchies (Figure 5).

Spatial distributions of discriminated assemblages show a general alignment along biogeographical units. *Alnus glutinosa*‐dominated assemblages agglomerate in the humid Alder carrs of Eastern Georgia (Nakhutsrishvili, 2013), whereas the *Pinaceae*‐dominated assemblages are predominantly located in montane to subalpine areas of the Lesser and Greater Caucasus. Assemblages dominated by mixed *Quercus* spp., *Carpinus* spp., and *F. orientalis* are situated at intermediate ranges. Those with high shares of BA of *Quercus* spp. are limited to lower and drier areas, while *F. orientalis*‐dominated assemblages are located at higher elevations. Interestingly, the five samples assigned to *Juniperus*‐*Pistacia* woodland have been clearly discriminated within HC_dA_ that are located in the semi‐arid areas of the southwest of the country (Nakhutsrishvili et al., 2021).

**FIGURE 6:**
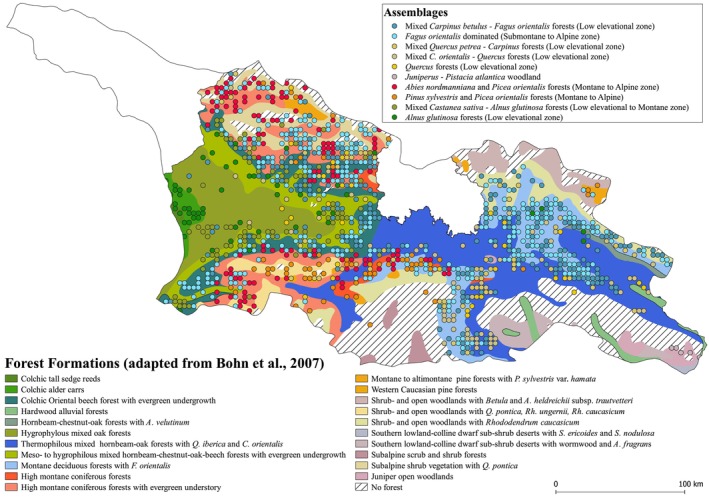
Schematic overview of spatial distributions of discriminated assemblages and main forest formations adapted from the vegetation‐type classification by Bohn et al. (2007). Assemblages are colored according to the respective palette of the four main classes of the cluster hierarchy (blue, beige, red/orange, and green for dominance of Fagus, Carpinus‐Quercus, Pinaceae, and Alnus‐Castanea, respectively).

To visually evaluate the spatial distribution of discriminated assemblages, we cut HC_dA_ at level II because 70% of the partitions are not partitioned further below level II, resulting in 10 clustered assemblages (Figure 4). Based on characteristic indicator species, relative BA distributions, and occupied elevational zones, we labeled each assemblage accordingly and mapped the respective sample location in relation to areas of forest vegetation‐type classifications according to Bohn et al. (2007) (Figure 6).

## 4 DISCUSSION

Parting from the assumption that genetic variation of co‐inhabiting species provides a signal that reflects site‐specific environmental determinants, we contrast the performance of a species‐neutral dissimilarity index (BC) with an index that considers interspecies genetic variation (dA) when used in unsupervised classification. Our findings indicate that incorporating interspecies phylogenetic distances in the quantification of dissimilarities results in more coherent and ecologically meaningful classifications of large‐scale forest inventory data with high β‐diversity.

The Mantel statistics indicate a significant positive correlation between the dissimilarity matrices obtained for each index (*r* = .613, with *p* = .0004), implying that essential patterns of variation among samples are maintained in the respective quantifications. However, frequency distributions and visual inspection of dissimilarities (Figure 4) show overall higher dissimilarity values based on BC, reflecting its' sensitivity to species turnover. Whereas the resulting cluster hierarchies maintain a certain level of agreement (cophenetic correlation = .511), the fact that a correlation of one signifies complete similarity suggests that the dissimilarity signal resulting from dA is not redundant. Clustering based on dA performs slightly better in preserving the original dissimilarities according to the respective cophenetic correlation coefficients of .492 and .442 for HC_BC_ and HC_dA_, respectively. Dendrogram topologies, cluster validation metrics (i.e., WSS of 344.123 and 112.917 for HC_BC_ and HC_dA_, respectively), and distributions of relative BA among assemblages indicate a higher degree of compactness, separation, and yield, generally more conceivable clustering results based on dA. Overall, HC_dA_ provided enhanced general interpretability and succeeded in discriminating clearly distinguished assemblages regarding compositional characteristics, that is, the *P. atlantica* and *Juniperus* woodlands of the semi‐arid lowlands of Southeastern Georgia. These results support the concept that an extension of variables considered for quantification of dissimilarity leads to a refined conception for diversity classification if genetic variation is considered and are in line with the findings of Hao, Ganeshaiah, et al. (2019) and other authors (Capelo, 2020; Pavoine & Ricotta, 2014; Ricotta & Pavoine, 2015).

As BC represents a “species‐neutral” diversity index sensu Chao et al. (2010), which assumes that all observed species contribute equally to overall diversity, and species turnover constitutes the predominant signal for discrimination, reflected by the significantly higher number of indicator species with frequencies of 100% (24 and 8 for HC_BC_ and HC_dA_, respectively). Conversely, as dA dissimilarity considers species as phylogenetic units, a complete species turnover does not necessarily result in maximum dissimilarity values between two sites because differences in abundance are weighted by the genetic proximity between species. Assuming that the genetic signal of co‐occurring species reflects niche occupation within given ecogeographical areas (Hawkins et al., 2014), the thus refined dissimilarity signal appears to respond to biogeographical gradients in a more interpretable manner.

The validity of the presented approach relies on precise measurements of tree diameters and accurate species identification in the field. While traditional forest science prioritized genus‐level information, growing emphasis on diversity‐related issues prompted forestry experts to be increasingly trained to provide accurate species identification. The related uncertainties are not design‐based issues but apply to all ecological surveys requiring botanical expert knowledge to ensure taxon detection and validate observations on species level (Lam & Kleinn, 2008; Roswell et al., 2021). Overall, only 2% of all cluster plot observations included individuals that were not identified to species level and had to be excluded. By considering only cluster plot observations of equal sample size (*m* = 3, 64% of all cluster plots) and the exclusion of subplots overlapping with the forest boundary (16%), our analyses are based on a subsample of the NFI data, representing 60% of all cluster plot observations. Hence, conclusions drawn from the presented results should consider, for example, that species exclusively occurring at forest boundaries are excluded. Potential limitations to validity arise from field sampling protocols, as overall subplot size, or sampling effects due to the nested subplot structure (with respective target DBH as inclusion criteria), may introduce bias to the quantification of dissimilarity (de Cáceres, Martín‐Alcón, et al., 2019). Accordingly, observed numbers of species should be regarded as proxies of true species richness, especially if nested sample plot designs are applied that are based on specific diameter thresholds (Lin et al., 2020). Overall species richness can be assumed to be higher with the contributions of smaller‐diameter trees being neglected (Corona et al., 2017). Aggregations to cluster plot level could potentially translate to generalization effects and the loss of information on site‐specific environmental factors on smaller scales. The resulting magnitude of impact on the presented results, however, is likely to vary according to forest type, topographical condition, and management regime (McRoberts et al., 2009).

With continuing advances in whole‐genome phylogenetics and functional genomics, information on phylogenetic diversity is continuously improving (Kling et al., 2018). Access to comprehensive and standardized phylogenetic mega trees to quantify species genetic relationships is readily available and their application to investigate variation in community compositions is becoming increasingly more common (Gilbert & Parker, 2022; Jin & Qian, 2022).

Our results are of relevance for a wide range of classifications of ecological entities according to conservation value, mapping of ecological communities, or other discriminative objectives. The method aligns standard canopy layer data from forest inventories with natural vegetation types according to Bohn et al. (2007), but harmonization with existing forest typologies is limited due to methodological differences, such as the structural vegetation layers considered and the abundance units recorded (e.g., Chytrý et al., 2020; Mucina et al., 2016). Investigating the degree to which the resulting clusters can be aligned with defined syntaxonomical units is an interesting area for future research, especially for the identification of diagnostic species from the shrub and herb layers to refine classifications and the development of practical assessment procedures.

The integration of genetic signals of forest communities during characterization has wide implications for respective approaches to classification. As a proxy indicative of the relationship between species composition and site conditions, interspecies genetic variation extends the scope of forest diversity mapping, management, and monitoring to account for alterations inconceivable by conventional compositional variables. Beyond the potential advantage of streamlining processes by applying unsupervised classification to large datasets, our approach is straightforward and can be readily replicated with comparable data, provided entities are assessed in a systematic manner. Ecological studies are frequently less systematic and constrained by temporal and spatial scales due to the dynamic nature of communities over time and space. This holds true to a lesser degree for assessments of woody species communities within the context of national forest monitoring systems, which are resampled in fixed intervals. Hence, from a practical point of view, the resulting network of observational studies provides a valuable framework for a systematic and recurring collection of ecological data, as additional costs and efforts can be embedded into existing structures. Extending the scope of study objectives to systematic assessments of a wider range of botanical and potentially zoological taxa could provide powerful and statistically robust data for analyses of organisms, structural components, and the interrelationships between them.

## 5 CONCLUSIONS

We present an approach to discriminate species diversity from NFI data of forest communities with high β‐diversity and species turnover. The novelty of the method lies in considering interspecies genetic variability for the quantification of diversity and subsequent classification using an unsupervised clustering algorithm on a country‐wide scale. We demonstrate that large‐scale forest inventory data can be classified in an ecologically meaningful manner based on mean basal area estimates per species and consideration of phylogenetic dissimilarity between samples. The thus obtained discrimination of species assemblages provides a differentiated picture of existing diversity patterns along expected biogeographical gradients without the need for additional assessments. This approach aligns with a biodiversity concept considering genetic diversity and could potentially be standardized for application to similar datasets, provided systematic data assessment is granted. The presented results should be considered as a step in evaluating to which extent large‐area forest inventory data could provide a backbone for extended biodiversity monitoring systems, as discrimination of woody species assemblages allows for systematic delineation of forest ecosystems if genetic variation is considered.

## AUTHOR CONTRIBUTIONS

**Alexander Wellenbeck:** Conceptualization (lead); data curation (lead); formal analysis (lead); investigation (lead); methodology (lead); validation (lead); visualization (lead); writing – original draft (lead); writing – review and editing (lead). **Lutz Fehrmann:** Conceptualization (supporting); formal analysis (supporting); methodology (supporting); supervision (supporting); visualization (supporting); writing – review and editing (supporting). **Hannes Feilhauer:** Formal analysis (supporting); methodology (supporting); writing – review and editing (supporting). **Sebastian Schmidtlein:** Methodology (supporting); software (supporting); writing – review and editing (supporting). **Bernhard Misof:** Methodology (supporting); supervision (supporting); writing – review and editing (supporting). **Nils Hein:** Conceptualization (supporting); investigation (supporting); methodology (supporting); project administration (lead); writing – original draft (supporting); writing – review and editing (supporting).

## CONFLICT OF INTEREST STATEMENT

The authors declare no competing interests.

## Supporting information


Figure S1:

Figure S2:

Figure S3:

Figure S4:

Appendix S1:

## Data Availability

The data that support the findings of this study are openly available in Dryad at https://doi.org/10.5061/dryad.wpzgmsbvv.
