# Supplementary figures and images for: Discriminating woody species assemblages from National Forest Inventory data based on phylogeny in Georgia
Source: Ecol Evol. 2024 Jul 23;14(7):e11569. doi: 10.1002/ece3.11569 (PMC11264350; doi:10.1002/ece3.11569)

Figure S1: Phylogeny of 87 observed species.

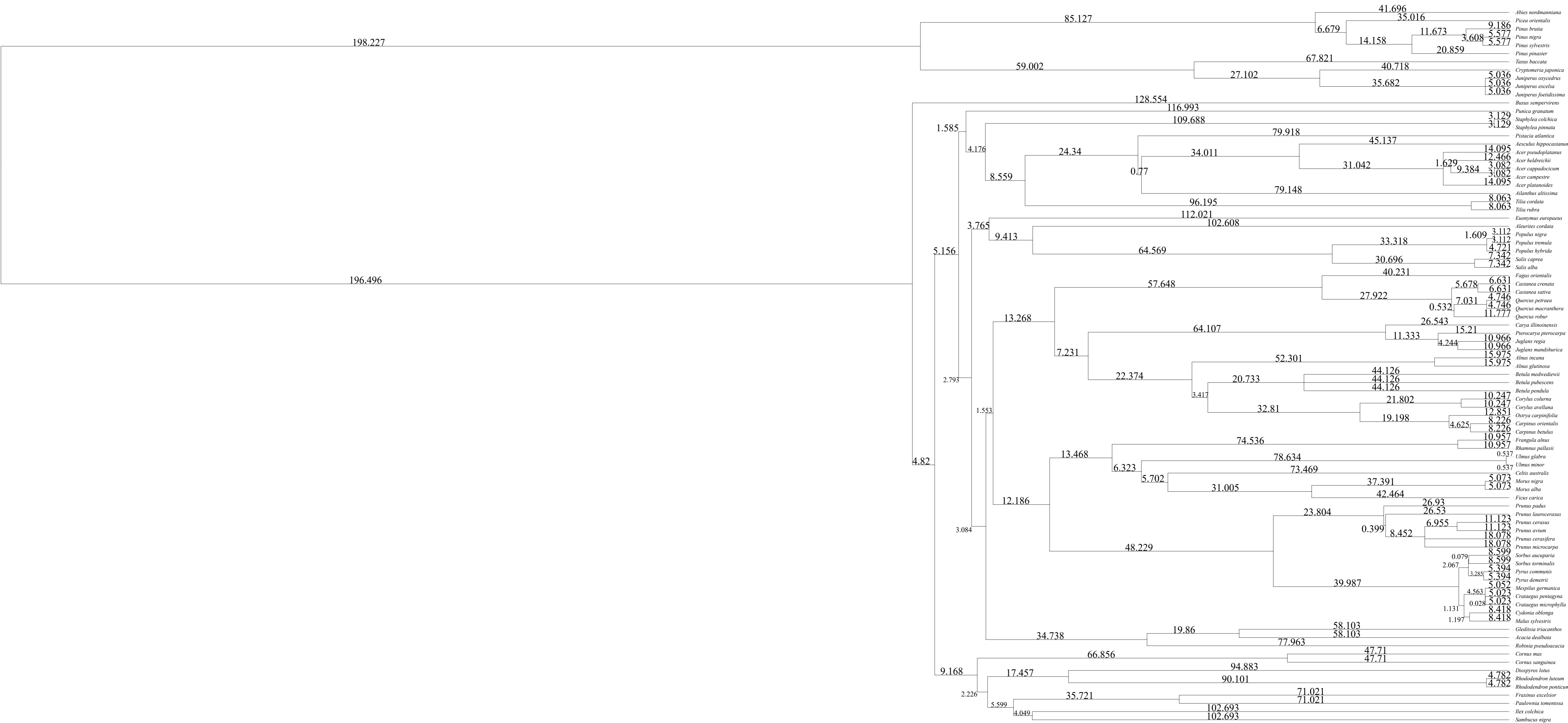

Supplement: Supplementary file 1 — Figure S1: [file ECE3-14-e11569-s002.pdf]
