# Supplementary material for: Discriminating woody species assemblages from National Forest Inventory data based on phylogeny in Georgia
Source: Ecol Evol. 2024 Jul 23;14(7):e11569. doi: 10.1002/ece3.11569 (PMC11264350; doi:10.1002/ece3.11569)

Figure S2: Frequencies of pairwise dissimilarities for compositional data between samples of the National Forest Inventory data of the Republic of Georgia based on Bray-Curtis (BC, a) and the discriminating Avalanche (dA, b) index based on the lower triangle of the respective dissimilarity matrices (1059 x 1059).

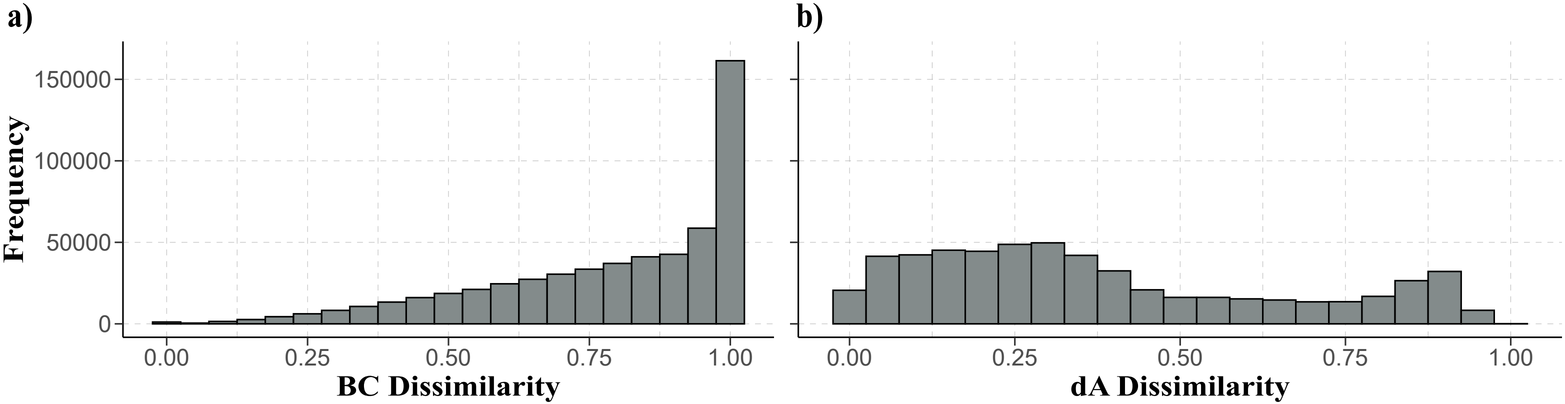

Supplement: Supplementary file 2 — Figure S2: [file ECE3-14-e11569-s003.pdf]
