# Supplementary material for: Discriminating woody species assemblages from National Forest Inventory data based on phylogeny in Georgia
Source: Ecol Evol. 2024 Jul 23;14(7):e11569. doi: 10.1002/ece3.11569 (PMC11264350; doi:10.1002/ece3.11569)
Supplement: Supplementary file 3 — Figure S3: [file ECE3-14-e11569-s005.pdf]

Figure S3: Synoptic table of ISOPAM hierarchical clustering based on Bray-Curtis dissimilarities for 1059 samples of the National Forest Inventory of the Republic of Georgia. The table shows an overview of the resulting assemblages (23) with respective indicator species frequencies (%) and levels of significance (*** ≙ p ≤0.001, ** ≙ p ≤0.01, * ≙ p ≤0.05).

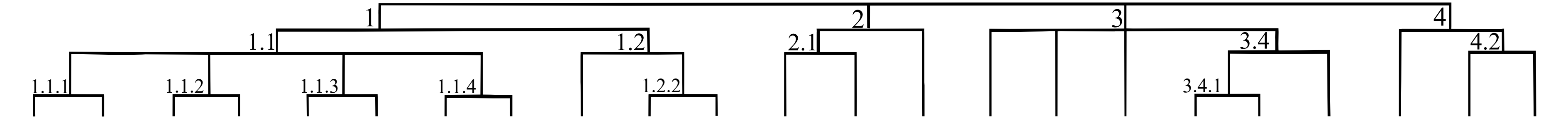

Assemblage	1.1.1.1	1.1.1.2	1.1.2.1	1.1.2.2	1.1.3.1	1.1.3.2	1.1.4.1	1.1.4.2	1.2.1	1.2.2.1	1.2.2.2	2.1.1	2.1.2	2.2	3.1	3.2	3.3	3.4.1.1	3.4.1.2	3.4.2	4.1	4.2.1	4.2.2
**n**	22	9	53	34	26	16	87	22	141	27	19	57	42	152	49	32	61	18	6	16	106	51	13
**S**	22	17	32	22	22	12	25	17	49	25	26	37	35	53	22	31	31	17	19	20	54	53	32
**Medoid**	1197	967	2376	2372	785	844	3021	3739	1470	2748	1322	4163	5052	1581	602	5321	3713	4260	4447	3511	2764	2016	2694
**Elevation [m asl]**	≤750 - 2000 m	1750 - ≥2000 m	≤750 - 2000 m	750 - ≥2000 m	≤750 - 2000 m	750 - ≥2000 m	750 - ≥2000 m	≤750 - 2000 m	750 - ≥2000 m	≤750 - 2000 m	≤750 - 2000 m	≤750 - 2000 m	≤750 - 2000 m	≤750 - ≥2000 m	1250 - ≥2000 m	≤750 - ≥2000 m	750 - ≥2000 m	750 - ≥2000 m	≤750 - 1750 m	1250 - 2000 m	≤750 - 1750 m	≤750 - 2000 m	≤750 - 1750 m
**Species**																							
Abies nordmanniana (Steven) Spach			2**	0**	50***	100***		0*	3***			0***	2*	5***	100***		62***				2***	2**	
Acer campestre L.							0***					54***	24**	14**	0*		0**						
Acer cappadocicum Gled.				6*		0*	9**	55***	33***	52***		37**		38***	4**	0***	10*	0*				4***	
Acer heldreichii subsp. trautvetteri (Medvedev) A.E.Murray	27**	56***					16*					0*	0*		24***	41***					1**		
Acer platanoides L.			25***	21*						33***											3*	0*	
Acer pseudoplatanus L.						44***	10*							1*									
Ailanthus altissima (Mill.) Swingle																						6**	
Alnus glutinosa (L.) Gaertn.			4***				9**		15*	4*	53**	7**	2***	5***	8*	0***				0*	87***	94***	62**
Betula medwediewii Regel	9*															6*							
Betula pendula Roth	32***	22*							1*							31***					0*		
Betula pubescens var. litwinowii (Doluch.) Ashburner & McAll.																16***		11*					
Carpinus betulus L.	27***	22*	94***		31***	12***	26***	100***	100***	96***	95**	91***	26***	95***	20***	0***	34***	6***			87***	45**	
Carpinus orientalis Mill.			4*	0**			0***		4***	0*		84***	93***	24***	0**	0*	3*				3***		
Carya illinoinensis (Wangenh.) K.Koch																						2*	
Castanea crenata Siebold & Zucc.											5*												
Castanea sativa Mill.				44**		0*	5***			41*	95***	4***	2***	14*	6**	0***	3***	0*		0*	85***		
Celtis australis subsp. caucasica (Willd.) C.C.Towns.																						2*	
Cornus mas L.												25***	17***								0*		
Cornus sanguinea subsp. australis (C.A.Mey.) Soó												12***	10**										15*
Corylus avellana L.										26**					0*		0*				21***	25***	
Corylus colurna L.									2*														
Crataegus microphylla K.Koch												21***	17***										
Crataegus pentagyna Waldst. & Kit. ex Willd.												18***	26***								0*		23**
Cryptomeria japonica (Thunb. ex L.f.) D.Don																					3***		
Cydonia oblonga Mill.																						2*	
Diospyros lotus L.																					12***	8*	54***
Euonymus europaeus L.											5*												
Fagus orientalis Lipsky	100***		100***	100***	100***	100**	100***	100***	99***	93**	100**	30***	7***		96***	28***	54**	6***			58**	8***	0***
Ficus carica L.																					5**		15**
Frangula alnus Mill.																						4**	
Fraxinus excelsior L.				0**	0*		0***					88***	36***	26***	0***	0**	2**				8*		
Gleditsia triacanthos L.																						6**	23***
Ilex colchica Pojark.											5*												
Juglans regia L.																						8**	23***
Juniperus excelsa subsp. polycarpos (K.Koch) Takht.																16***							
Juniperus foetidissima Willd.																6***							
Juniperus oxycedrus L.																9***							
Malus sylvestris subsp. orientalis (Uglitzk.) Browicz							0*															14**	23*
Morus alba L.																						6**	15**
Morus nigra L.																					6**		31***
Ostrya carpinifolia Scop.														2*									
Picea orientalis (L.) Peterm.				6*	100***				10***	4*	0*	4***	2***	13**	82***		100***			100***	11**	2***	0*
Pinus nigra J.F.Arnold																6*							
Pinus pinaster Aiton																						2*	
Pinus sylvestris var. hamata Steven				0*			1***		5**								46***	100***	100***	100***	4**	2*	
Pistacia atlantica Desf.																9***							
Populus hybrida E.H.L.Krause																						6**	
Populus nigra L.									4**														
Populus tremula L.		89***					1**					0**	0*	14*						81***	2**		
Prunus avium (L.) L.							1**		18**	26*				18**									38**
Prunus cerasifera Ehrh.														5*							6*		23**
Prunus laurocerasus L.											26***			0**							23***		
Prunus microcarpa C.A.Mey.																	7**						
Prunus padus L.																3*							
Pterocarya pterocarpaÂ (Michx.) Kunth ex Dippel																						8***	
Punica granatum L.																						2*	
Pyrus communis subsp. caucasica (Fed.) Browicz				0*	0*		2***		27***					27***	2**		3**		83***				38*
Quercus macranthera Fisch. & C.A.Mey. ex Hohen.									10***														
Quercus petraea subsp. polycarpa (Schur) Raus	0***		15*	6**	0***	0**	0***		20*			82***	90***	88***	2***	3***		0**			15***	2***	
Quercus robur subsp. imeretina (Steven ex Woronow) Menitsky												7*				9*					5*		
Rhamnus pallasii Fisch. & C.A.Mey.													2*										
Rhododendron ponticum L.																					2*		
Robinia pseudoacacia L.																					9***		92***
Salix alba L.																						4*	
Salix caprea L.	32***						1*					0*				25**	26***				1**		
Sambucus nigra L.																					16***	12**	
Sorbus aucuparia L.	18*	100***							1*							31***							
Sorbus torminalis (L.) Crantz			15*											22***	0*						2*	0*	
Staphylea colchica Stev.																					3**		
Staphylea pinnata L.											5*												
Tilia rubra subsp. caucasica (Rupr.) V.Engl.							3***			100***	63***	39**	5**	34***		0***	10*	0*		0*	32*	6**	
Ulmus glabra Huds.													0*								14*		
Ulmus minor Mill.																						16*	
Vernicia fordii (Hemsl.) Airy Shaw																					5**		
