# Supplementary material for: Discriminating woody species assemblages from National Forest Inventory data based on phylogeny in Georgia
Source: Ecol Evol. 2024 Jul 23;14(7):e11569. doi: 10.1002/ece3.11569 (PMC11264350; doi:10.1002/ece3.11569)
Supplement: Supplementary file 5 — Appendix S1: [file ECE3-14-e11569-s004.pdf]

Supplement 5: Description of indicator species and group characteristics of the resulting ISOPAM partition based on the discriminating Avalanche below level I.

At level II of the hierarchical clustering based on the discriminating Avalanche index (HC_{dA}) only the branches representing Fagus and Carpinus-Quercus dominated samples are further partitioned, whereas the Pinaceae and Alnus-Castanea dominated branches remain undivided below level II, resulting in two final groups, respectively. The branches of the Fagus dominated class, 1.1 and 1.2, are mainly distinguished by differences in significant frequencies of gymnosperms, *A. cappadocicum*, *C. sativa*, *F. excelsior*, *T. rubra* and *Q. petrea*. In 1.1, presence of the latter indicator species is highly significant, whereas in 1.2 the frequencies of gymnosperms are generally higher. 1.1 is partitioned into three branches, with branch 1.1.1 being further divided into two final assemblages, of which 1.1.1.2 contains a relatively large share of 122 (12%) samples. Assemblage 1.1.1.2 consists mainly of samples that contain *C. betulus* (98%) and *F. orientalis* (87%), with an absence of *A. nordmanniana* (0%). In contrast, in 1.1.1.1 78% of all samples contain *Q. petrea,* 39% *F. excelsior,* and 30% *Sorbus torminalis* (L.) Crantz, all of which are completely absent in assemblage 1.1.1.2. 100% of samples in branch 1.1.2 contain *T. rubra* and *C. betulus* with a presence of *Acer pseudoplatanus* L. in 56% of all samples. Group 1.1.3 represents mixed *F. orientalis* (89% at $p \leq 0.01$) and *C. sativa* (89%) assemblages with *A. glutinosa* (58%). Samples in assemblage 1.1.2 (100%) all contain *T. rubra* and significant shares of *Acer cappadocicum* Gled. (56%) and *Acer platanoides* L. (36%). Branch 1.2 is partitioned into three branches at level II of which 1.2.1 and 1.2.2 contain three and two assemblages at level IV, respectively. The third branch 1.2.3.0 is not divided further. The three assemblages of branch 1.2.1 are characterized by the presence of *A. nordmanniana* and *P. orientalis*. 59% of all samples in 1.2.1.1 contain *A. nordmanniana* and 71% *P. orientalis,* with 100% of samples containing *F. orientalis*. In contrast, in assemblages 1.2.1.2 and 1.2.1.3 few samples contain *A. nordmanniana* (2% and 1%, respectively), but significantly higher shares of *C. betulus*, with 48% and 99% of all samples, respectively. *Tilia rubra subsp. caucasica* (Rupr.) V.Engl. is present in 38% of all samples in 1.2.1.3, whereas it is observed in only 10% of the relatively large group 1.2.1.2 (15% of all samples, $n = 163$). Gymnosperms are entirely absent from 1.2.2.1 and 1.2.2.2. The relatively small assemblage 1.2.2.2 ($n = 3$) is characterized by the complete absence of *C. betulus* and *F. orientalis* (not significant at $p \leq 0.05$) and represent samples that contain *A. pseudoplatanus* (100%), *Betula medwediewii* Regel. (100%) and *Sorbus aucuparia* L. (67%). All samples in this assemblage are located above 2000 m asl. The separate assemblage 1.2.3 of branch 1.2 displays a presence of *Pinus sylvestris* var. *hamata* Steven in 100% of all samples, whereas *C. betulus* and *F. orientalis* are entirely absent (not significant at $p \leq 0.05$).

The Carpinus-Quercus dominated class is divided into two branches of which only 2.2 is partitioned into two additional branches. At level IV branch 2.2.1 is divided into two assemblages. No indicator species

is highly significant for assemblage 2.1.0.0. However, the number of total species present is comparably high (41) and 91% of all samples contain *C. betulus* (with $p \leq 0.05$) and 96% *Q. petrea* (not significant). Assemblages 2.2.1.1. and 2.2.1.2 consist of samples with high frequencies of *C. orientalis*, and *Q. petrea* with 97% and 90% and 78% and 90%, respectively. These assemblages are distinguished by the absence of *F. orientalis* (not significant) and a high frequency of *F. excelsior* (60%) in 2.2.1.2. Indicators in 2.2.2.0 are *A. campestre* (83%), *C. orientalis* (67%), *F. excelsior* (89%) and *Q. petrea* (78%), with a highly significant absence of *A. glutinosa* and *C. sativa*. All samples in 2.2.2.0 are situated below 1250 m asl. In assemblage 2.3.0.0, 81% of all samples contained *Q. petrea*, with the absence of *P. orientalis* and *T. rubra* being highly significant. 72% of all samples within this assemblage are located below 1250 m asl. The smallest assemblage containing only 5 samples (and four species in total) is 2.4.0.0. All samples are located below 750 m asl and the group is characterized by *Juniperus excelsa* subsp. *polycarpos* (K. Koch) Takht. (100%), *Juniperus oxycedrus* L. and *Pistacia atlantica* Desf. (60%, respectively).

In the Pinaceae-dominated class, assemblages 3.1.0.0 and 3.2.0.0 are distinguished by the frequencies of *A. nordmanniana* (89% and 32%, respectively), *F. orientalis* (89% and 25%, respectively), and *P. orientalis* (84% and 68%, respectively). *P. sylvestris* is entirely absent (not significant at $p \leq 0.05$) from 3.1.0.0, but present in 60% of all samples in 3.2.0.0. The two branches of the Alnus-Castanea dominated class are characterized by high frequencies of *A. glutinosa* as indicator species with 89% and 96% for assemblage 4.1.0. and 4.2.0.0, respectively. 4.1.0.0 is characterized by 82% of all samples containing *C. orientalis*, and 87% *C. sativa*, both of which are absent in 4.2.0.0. In addition, *F. orientalis* is present in 51% of all samples in 4.2.0.0, whereas only in 7% of all samples in assemblage 4.2.0.0.
