# Supplementary material for: Discriminating woody species assemblages from National Forest Inventory data based on phylogeny in Georgia
Source: Ecol Evol. 2024 Jul 23;14(7):e11569. doi: 10.1002/ece3.11569 (PMC11264350; doi:10.1002/ece3.11569)
Supplement: Supplementary file 4 — Figure S4: [file ECE3-14-e11569-s001.pdf]

Figure S4: Synoptic table of ISOPAM hierarchical clustering based on discriminating Avalanche dissimilarities for 1059 samples from the National Forest Inventory of the Republic of Georgia. The table shows an overview of the resulting assemblages (20) with respective indicator species frequencies (%) and levels of significance (*** ≙ p ≤0.001, ** ≙ p ≤0.01, * ≙ p ≤0.05).

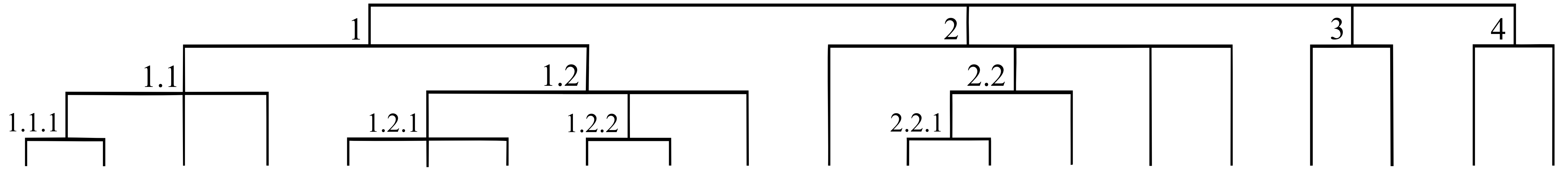

Subcluster	1.1.1.1	1.1.1.2	1.1.2	1.1.3	1.2.1.1	1.2.1.2	1.2.1.3	1.2.2.1	1.2.2.2	1.2.3	2.1	2.2.1.1	2.2.1.2	2.2.2	2.3	2.4	3.1	3.2	4.1	4.2
n	54	122	25	38	41	163	89	14	3	14	53	32	20	18	32	5	128	63	91	54
S	39	53	26	33	22	36	38	17	6	26	41	34	29	26	32	4	34	35	53	51
Medoid	3656	1801	2943	1845	3292	2281	1862	3930	913	565	1573	5117	2931	5248	4666	5319	3165	2010	2885	2014
Elevation [m asl]	≤750 - 2000 m	≤750 - >2000 m	≤750 - 1749 m	≤750 - 2000 m	1000 - >2000 m	≤750 - >2000 m	1250 - 2000 m	1500 - >2000 m	>2000 m	≤750 - 2000 m	≤750 - 1750 m	≤750 - 1250 m	≤750 - 1500 m	≤750 - 1250 m	≤750 - 2000 m	≤750 m	≤750 - >2000 m	1000 - >2000 m	≤750 - 2000 m	≤750 - 1500 m
**Species**																				
Abies nordmanniana (Steven) Spach	4*	0***		0**	59***	2***	1***				6	3*					80***	32***	3***	0***
Acer campestre L.					0*	1***					23	31***	50***	83***			2**			
Acer cappadocicum Gled.	48***	32**	56***		7*		31*	0		0*	43	6*	50**				11**	8**		0***
Acer heldreichii subsp. trautvetteri (Medvedev) A.E.Murray						13**	1**	79									20***		2*	0*
Acer platanoides L.			36***				15*							33					2*	0*
Acer pseudoplatanus L.					15**				100											
Ailanthus altissima (Mill.) Swingle	4*																			6**
Alnus glutinosa (L.) Gaertn.	2***		4*	58***		12***	11**	0*			6	3**		0***	3**		11***	3***	89***	96***
Betula medwediewii Regel					5*				100											
Betula pendula Roth								43										14***		
Betula pubescens var. litwinowii (Doluch.) Ashburner & McAll.								29		14*										
Carpinus betulus L.	96***	98***	100***	97***	24***	48***	99***	7**	0***		91*	34***	95**	94	34***	0**	31***	25***	82***	
Carpinus orientalis Mill.	28**		0*		0**	1***	2***				40	97***	90***	67***	50***		2***	3*	3**	
Castanea crenata Siebold & Zucc.			4*																	
Castanea sativa Mill.	9*		40*	89***	7*			0*		0*	8	3**		0***			6***	0***	87***	
Celtis australis subsp. caucasica (Willd.) C.C.Towns.												3*								
Cornus mas L.						0**					11	16**		44**			0*			
Cornus sanguinea subsp. australis (C.A.Mey.) Soó											6	9**	10*	17***						
Corylus avellana L.			24**										25*				1***		21***	20**
Crataegus microphylla K.Koch	9*					0**					9	22***		22***			0*			
Crataegus pentagyna Waldst. & Kit. ex Willd.						0**						22***	30***		19***		0*			
Cryptomeria japonica (Thunb. ex L.f.) D.Don																			2*	
Diospyros lotus L.						0*											0*		16***	17***
Fagus orientalis Lipsky	98***	87***		89**	100***	99***	100***	29***			36	16***		22***	3***	0**	89***	25***	51***	7***
Ficus carica L.																			7***	
Frangula alnus Mill.																			2*	
Fraxinus excelsior L.	39***			3*	0**	2***					51	47***	60***	89***	31*		1***	2***	4**	
Gleditsia triacanthos L.																				7***
Ilex colchica Pojark.				3*																
Juglans regia L.																				11***
Juniperus excelsa subsp. polycarpos (K.Koch) Takht.																100***				
Juniperus foetidissima Willd.																40***				
Juniperus oxycedrus L.																60***				
Malus sylvestris subsp. orientalis (Uglitzk.) Browicz		9*				1**											1*			17***
Mespilus germanica L.														6						
Morus alba L.																				4*
Morus nigra L.																				13***
Ostrya carpinifolia Scop.															6**					
Picea orientalis (L.) Peterm.	6***	5***	4*		71***	5***	13*	0*				3**		0**	0***		84***	68***	8***	0***
Pinus nigra J.F.Arnold																		6***		
Pinus sylvestris var. hamata Steven		3**				2***	3**			100***								87***		2*
Pistacia atlantica Desf.												6**				60***				
Populus hybrida E.H.L.Krause	4*														6*					
Populus tremula L.										43***							14*	27***	2*	0**
Prunus avium (L.) L.	22**	16*	28**		0*	3***														
Prunus cerasifera Ehrh.														22						
Prunus laurocerasus L.																	0**		29***	
Prunus microcarpa C.A.Mey.																		8**		
Prunus padus L.								7												
Pterocarya pterocarpaÂ (Michx.) Kunth ex Dippel																				9***
Punica granatum L.												3*								
Pyrus communis subsp. caucasica (Fed.) Browicz	31***	29***				1***									28*		2***			
Quercus macranthera Fisch. & C.A.Mey. ex Hohen.		8**																8*		
Quercus petraea subsp. polycarpa (Schur) Raus	78***				0***	4***	13***				96	78***	90***	78***	81***		9***		15**	6***
Quercus robur subsp. imeretina (Steven ex Woronow) Menitsky				8*								9*			16***					
Rhamnus pallasii Fisch. & C.A.Mey.												3*								
Robinia pseudoacacia L.						0**											0*		11***	22***
Salix alba L.																				6**
Salix caprea L.								29	67*									25***		
Sambucus nigra L.																	0*		21***	9*
Sorbus aucuparia L.							0*	71	67***											
Sorbus torminalis (L.) Crantz	30***										26			22	19*				1*	
Staphylea colchica Stev.																			3**	
Taxus baccata L.						2*														
Tilia rubra subsp. caucasica (Rupr.) V.Engl.	35*		100***	37*	5**	10***	38***	0***			36			67	0***			2***	35**	11*
Ulmus minor Mill.			24**																	15*
Vernicia fordii (Hemsl.) Airy Shaw																			4	7
